# Using individual-level stratification as an approach to integrating social inequalities into the burden of disease

**DOI:** 10.1093/eurpub/ckac129.400

**Published:** 2022-10-25

**Authors:** CM Baravelli, TA Eikemo, ST Klitkou, JM Kinge, BM Clarsen, AK Bølling, M Balaj, AKS Knudsen

**Affiliations:** 1 Department of Disease Burden, Norwegian Institute of Public Health, Bergen, Norway; 2 Centre for Global Health Inequalities Research, Norwegian University of Science and Technology, Trondheim, Norway

## Abstract

Substantial social inequalities in almost all non-fatal and fatal health outcomes are one of the most consistent and universal epidemiological findings. Therefore, monitoring social inequalities in health is considered a key priority for researchers and policy makers. The Global Burden of Disease Injuries, and Risk Factors Study (GBD) is the most comprehensive worldwide observational epidemiological synthesis of data to date. However, currently, the GBD Study does not include the potential to stratify associated metrics, such as the disability-adjusted life years metric, by different socioeconomic factors, such as education or income level. Although The GBD Study does include the Socio-Demographic Index, this measure is only useful when comparing between, and not within, countries or regions. We conducted a Cox regression analysis using a national longitudinal prospective cohort study design and registry-based data linked at the individual-level. We stratified on educational groups and investigated cause-specific mortality rates over a 30-year period, adjusting for age, sex and 5-year age cohorts. We also calculate years of life lost (YLLs) stratified by educational groups, standardised by age, and presented for specific years - to investigate trends over time. We discuss the benefits and limitations of this “individual-level” stratification approach as one possible solution to the integration of social inequalities into the GBD study or when using a burden of disease framework approach more generally.

